# Genetic diversity of fluorescent protein genes generated by gene duplication and alternative splicing in reef-building corals

**DOI:** 10.1186/s40851-015-0020-5

**Published:** 2015-07-21

**Authors:** Shiho Takahashi-Kariyazono, Yoko Satta, Yohey Terai

**Affiliations:** Department of Evolutionary Studies of Biosystems, SOKENDAI (The Graduate University for Advanced Studies), Shonan Village, Hayama, Japan

**Keywords:** Corals, Fluorescent protein, Gene duplication, Alternative splicing

## Abstract

**Introduction:**

Reef-building corals (Scleractinia) exhibit various colors, of which fluorescent proteins (FPs) are a major determinant. Gene duplication is considered a major mechanism in the generation of the *FP* gene family and color diversity. Examining gene duplication events and subsequent evolution may improve our understanding of *FP* gene family diversity.

**Results:**

We isolated a novel *FP* gene family from one individual of *Montipora* sp., which we named *monGFP* (*GFP* gene from *Montipora* sp.). This gene family consists of at least four genes that produce at least six different cDNA sequences. The sequences were categorized into two types based on the length of cDNA; this difference is attributed to alternative splicing. Although the amino acid sequences were different, the emission spectra of the *monGFP* variants were nearly identical (518–521 nm). In addition to this gene family, we isolated ten paralogous *AdiFP10* (*Adi-Fluorescent protein-10* gene from *Acropora digitifera*) sequences from cDNA of two *Acropora* species, *A. digitifera* and *A. tenuis*. Based on our phylogenetic analysis, five sequences from *A. digitifera* and four sequences from *A. tenuis* appeared to be in a different cluster from *AdiFP10*, suggesting a new *FP* gene cluster. The *FP* sequences were likely to have been generated independently in each species or generated by gene duplications in the ancestral lineage of *Acropora*, followed by extensive gene conversion within each species.

**Conclusion:**

Our results clarify a part of the diversification process of *FP* genes during the evolutionary history of *Montipora* and *Acropora* species. Our analyses of monGFP indicate that FPs translated from different splicing variants and gene copies have evolved without changes in the function of fluorescence, and gene copies have been evolved under purifying selection. On the other hand, *AdiFP10* paralogs and other *RFP* genes in *Acropora* species may have diversified their functions. Identification of conserved and divergent modes of evolution after the duplication of *FP* genes may reflect variation in the biological roles of different FPs.

**Electronic supplementary material:**

The online version of this article (doi:10.1186/s40851-015-0020-5) contains supplementary material, which is available to authorized users.

## Introduction

In tropical shallow oceans, reef-building corals (Scleractinia) exhibit various colors. Fluorescent proteins (FPs) are a major color determinant in corals [1–3]; indeed, the pigments in different color morphs have been shown to be correlated with copy number variation in an *FP* gene [4]. FPs are excited by environmental light and emit a longer wavelength of fluorescent light than the excitation light [5], and this fluorescent light can be perceived as “color”. FP color property is determined by amino acid sequences of FPs; in this sense, the evolution of *FP* genes represents the evolution of colors in corals [6].

The initial discovery and determination of the structure of FP was first studied in the crystal jelly, *Aequorea victoria* (synonym *A. aequorea*) [7, 8]. This protein, called green fluorescent protein (GFP), is excited by blue light (wavelengths shorter than 470 nm) and emits green fluorescence (maximum emission light: λemiss = 509 nm) [9]. Since the discovery of FP in the crystal jelly, *FP* genes have been cloned from anthozoa [10], marine crustacean copepods [11], and deuterostome chordate amphioxus species [12–14]. The emission of florescence with longer wavelengths than excitation light is the most useful molecular tool for *in vivo* imaging techniques [15].

A variety of FPs has been detected in anthozoan species, with emission spectra in the visible light range. FPs are classified into four groups based on the color of light detected: cyan (CFP), green (GFP), yellow (YFP), and red (RFP) [16, 17]. Since blue/purple non-fluorescent chromoprotein is in the same phylogenetic cluster with other *FP* genes, it is also classified as an *FP* gene family member [16]. The origin of *FP* genes pre-dates the divergence of coral families, and may have occurred as early as the Jurassic period [2]. During coral evolution, diversification of fluorescent colors has occurred across species and FPs of the same fluorescence class have emerged repeatedly and independently; for example, CFPs, YFPs, and RFPs have evolved several times in different lineages [17].

In addition to fluorescent emission, many roles of FPs have been proposed; they are thought to be essential for viability, and may have photoprotective and antioxidant roles [4, 18–20]. However, detailed biological roles of FPs in corals remain unclear as the functional mechanisms have not been elucidated.

Gene duplication is a major source of genetic variation and can lead to neofunctionalization of the genes [21, 22]. Neo-functionalization is a mechanism under which one copy of a duplicated gene retains the original function, leaving the other copy free from purifying selection and able to acquire new functions [22]. Beside the acquisition of a new function, gene duplication can also increase the transcription level by multiple copy number of genes, as is seen in the gene family that encodes ribosomal RNA [23]. In *FP* genes, gene duplication is considered to be a major mechanism of generating *FP* gene family and color diversity [2]. For example, ten *FP* genes have been isolated from the both genome and transcriptome of *Acropora digitifera* [24, 25]. Genomic analysis identified two regions, each of which consists of multiple copies of different *FP* genes [25]. Different copies of *amilFP597* genes are present in head-to-tail tandem arrangements in the *Acropora millepora* genome [4]. The phylogenetic analysis of available *FP* gene sequences showed that *FP* gene duplication and the subsequent acquisition of various fluorescent colors has occurred multiple times, indicating repeated evolution of FP colors [17]. In addition to the acquisition of different colors, copy number variation of *amilFP597* is correlated with red pigment concentration [4], indicative of increase in FP of the same color. The study of gene duplications and subsequent genetic diversification process of *FP* genes is important for understanding *FP* gene family diversity.

Alternative splicing, in which mature mRNAs are generated from a single pre-mRNA transcript, is another mechanism that may generate variation of protein functions. Alternative splicing increases protein diversity because the mature mRNAs sometimes encode proteins with subtly different or opposing functional domains [26]. However, alternative splicing of *FP* transcripts has not been reported, to our knowledge.

In this study, to evaluate the genetic diversification of the *FP* gene family, we analyzed gene duplication events and alternative splicing of *FP* genes in Scleractinia. We isolated novel *GFP* genes from *Montipora* sp., and identified a role of both gene duplication and alternative splicing in *FP* diversification. In addition to the *GFP* family, novel paralogs of an *RFP* gene were also identified in *Acropora* species. Our results shed new light on *FP* gene diversification during the evolutionary history of corals.

## Materials and Methods

### Specimens and species identification

*Montipora* sp. was acquired via an aquarium trade. The genus was predicted based on the sequence of the mitochondrial *COI* region (Additional file 1: Fig. S1) using the primers and method described in a previous study [27]. The sequence of *COI* region was aligned by using Genetyx (ver. 16.0.0), and subjected to a phylogenetic analysis (neighbor-joining method [19]) with 1,000 bootstrap replications. Under permits from the Aquaculture Agency of Okinawa Prefecture (number 26–9), parts of an *Acropora digitifera* and *A. tenuis* colony were collected and subsequently maintained in an aquarium at the Sesoko Station, Tropical Biosphere Research Center, University of the Ryukyus. The two species were identified based on morphology.

### DNA and RNA extraction

Genomic DNA was extracted from adult coral tissues of each species using the DNeasy Blood & Tissue Kit (Qiagen, Hilden, Germany). Total RNA was extracted from adult tissues and larvae of each species using the RNeasy Mini Kit (Qiagen).

### Isolation of novel *FP* cDNA

cDNAs were synthesized using a PrimeScript II 1st Strand cDNA Synthesis Kit (Takara, Shiga, Japan) from total RNA of *Montipora* sp.. An *FP* partial cDNA was cloned by RT-PCR using degenerate primers RFP_F2 (5’-ATGGAAGGGTSTGTCGATGG-3’) and RFP_R (5’-TKTTCTACCAGTYGCCATTTCTG-3’), and full-length cDNAs were isolated from the RNA of *Montipora* sp. by 5’ rapid amplification of cDNA ends (RACE) and 3’ RACE using the nested primers M5_RACE_F1 (5’-CAGTTCCACAGTACTTACAAGGCAAAGAC-3’), M5_RACE_R1 (5’-CATCAGGGCATGAGTTCTTGAAGTAGTC-3’), M5_RACE_F2 (5’-CGAAAGAGATGCCAGACTTCCACTTC-3’), and M5_RACE_R2 (5’-CAACTATGCCTCTGGGATATTTAGTGATG-3’), and a GeneRacer Kit (Invitrogen, Carlsbad, CA, USA). PCR was performed using the GeneAmp PCR System 9700 (Applied Biosystems, Carlsbad, CA, USA). All positions of primers are described in Fig. S2. The PCR reaction condition for partial cDNA was a denaturation step for 3 min at 94 °C, followed by 30 cycles of denaturation for 1 min at 94 °C, annealing for 1 min at 55 °C, and extension for 1 min at 72 °C. The 5’ and 3’ RACE procedures were performed with two rounds of nested PCR using M5_RACE_F1 and M5_RACE_R1 for the first round, and M5_RACE_F2 and M5_RACE_R2 for the second round. The first and second PCRs were performed under the same condition, consisting of a denaturation step for 3 min at 94 °C, followed by 30 cycles of denaturation for 1 min at 94 °C, annealing for 1 min at 65 °C, and extension for 3 min at 72 °C. The novel *FP* cDNA was amplified using M5_F1 (5’-ATGCAAGCCAACAAATGCGCAA-3’) and M5_R1 (5’-CAAGGAGCTTGCAGATGCAGAAG-3’) primers and cDNA of *Montipora* sp. as a template. The PCR condition for the *FP* cDNA consisted of a denaturation for 3 min at 94 °C, followed by 35 cycles of denaturation for 1 min at 94 °C, annealing for 1 min at 55 °C, and extension for 1 min at 72 °C. The PCR product was purified for direct sequencing or cloned into the T-Vector pMD20 vector (Takara), and the sequences were determined using the Applied Biosystems Automated 3130 Sequencer.

### Determination of the exon-intron structure of novel *FP* genes

The novel *FP* gene was amplified using M5_TC_LF (5’-GCAAACCGTGTGCTTGGATTCAC-3’) and M5_TC_LR (5’-CTTCCCACCAGTTGCCATTTCTG-3’) primers from the genomic DNA of *Montipora* sp. The PCR condition for the genomic *FP* gene sequence was denaturation for 3 min at 94 °C, followed by 30 cycles of denaturation for 1 min at 94 °C, annealing for 1 min at 65 °C, and extension for 10 min at 72 °C. The sequences of the whole intron 1, both ends of the other introns, and all exons were determined from the PCR product using M5eF1 (5’-CAACAAATGCGCAAAKAAGGCAAAC-3’), M5eR1 (5’- ACAGATCGCTTGATGCCTCAGAAG-3’), M5eR1_2 (5’- GGAATTTCAGGTCCATTTTGC-3’), M5eF2 (5’-TTGTATTACTTCTGAGGCATCAAG-3’), M5_RACE_R2 (5’-CAACTATGCCTCTGGGATATTTAGTGATG-3’), M5eF3 (5’-GGTTGACTATTTCAAGAACTCATG-3’), M5eR2 (5’- CATCACAGGTCCATTAGCAGGAAAG-3’), M5_TC_LR, and M5_GT_LR as sequencing primers. Partial intron 1 sequences between exon 1 and the additional 105 bp sequence (see results and discussion) were amplified using primers M5eF1 and M5eR1 from the genomic DNA of *Montipora* sp.. PCR products were cloned into T-Vector pMD20 and the sequences were determined using M5eF1, M5eR1, and M5i1_2_4F (5’-CGTCAAGATCCAGATTAACCTGT-3’) for all intron 1 sequences; M5i1L770 (5’-TGCTGAGACGGAAAATCTCAATGCT-3’) and M5i1-770R (5’-AGTCTGTTCTTAATAGCATTGAGA-3’) for *i1_1250* (the name of intron 1 type differed by the length, see results and discussion); M5i1-1300R (5’-TAGATCTCAGGTTACGTAATTCTGA-3’) and M5i1_3R (5’-CGACAGCTGACGTGCGATCTAAC-3’) for *i1_1800*; M5i1_2R (5’-AGTTAAGACATACGACAATAACAAAACGCA-3’), M5i1L1700 (5’- TTAGCTCGTAGTGCCGTGGAAAGTTAG-3’), and M5i1-1700R (5’-AGTCACGCTCCATTAACTACGT-3’) for *i1_2100*; and M5i1_1R (5’-AAAGACATTATTAGATCTGTTACGTAATTT-3’), M5i1-2400 F (5’-TCTCAGTATAAAAGGTCCAAAGTC-3’), and M5i1-2400R (5’-TTAAAGAACTTAGTGTTCACTAG-3’) for *il_2800*. The rest of intron 1 sequences (downstream part) were amplified from the genomic DNA of *Montipora* sp., using the primer pairs of M5eR1_2 and specific to each intron type M5i1-2400 F, M5i1-1700 F, and M5i1-770. The rest of the intron 1 sequences were determined from the PCR product using M5i1_2_4F and M5eR1. The whole intron 1 sequences were amplified from the genomic DNA of *Montipora* sp., using a primer pair of M5eF1 and M5eR1_2. DNA fragments of each intron type from exon 1 through to intron 1, and intron 1 through to exon 3 were amplified from the genomic DNA of *Montipora* sp., using the primer pairs of M5F1 and specific to each intron type M5i1-2400R, M5i1-1700R, and M5i1-770R for exon 1, and those of M5_RACE_R2 and specific to each intron type M5i1-2400 F, M5i1L1700, and M5i1L770 for exons 2 and 3. The sequences of intron 2 and partial sequences of exon 1 and exon 3 were determined using the M5i1-2400R, M5i1-1700R, M5i1-770R, and M5i2LF (5′-CGAATAGCTCAAGTTGAACTCAAGCGA-3′) primers.

### Determination of paralogs of *AdiFP10* sequences from *Acropora* species

Complementary DNAs were synthesized using a PrimeScript II 1st Strand cDNA Synthesis Kit (Takara) from total RNA of *A. digitifera* and *A. tenuis*. A set of primers AdiFP10_F2 (5’-TACGGAAACAGGGTCTTCACTG-3’) and AdiFP10_R1 (5’-TGTTCTGCCAGTTGCCATTTCTG-3’) were designed based on *AdiFP10* (accession number: BR000970). Using these primers and cDNA of *A. digitifera* and *A. tenuis* as templates, *FP* cDNAs were amplified. PCR products were cloned and the sequences were determined. The sequences were aligned using Genetyx (ver. 16.0.0). In order to remove PCR errors, cDNA sequences appeared at least two times were used for phylogenetic analysis.

### Phylogenetic and evolutionary analysis

The coding sequence of the novel *FP* gene was aligned with *FP* genes from other Scleractinia species by using Genetyx (ver. 16.0.0) and ClustalW [28], and subjected to a phylogenetic analysis (maximum likelihood method [29]) with 1,000 bootstrap replications. Sites with gaps or missing data were eliminated from the whole sequences used in following analyses. dN/dS values were calculated by using MEGA 5 [29]. The nucleotide sequences were deposited in GenBank under accession numbers LC029006 – LC029029.

### Recombinant FP protein and spectroscopy

*FP* cDNAs were cloned into a pCold I expression vector (Takara), and then transformed into the BL 21 *Escherichia coli* strain (Takara). Each clone was grown in 30 ml of LB medium overnight, and the recombinant protein was extracted by sonication and centrifugation. The S2 sequence in the expression vector was constructed from the S3 sequence by in vitro mutagenesis. *In vitro* mutagenesis of *FP* was performed by extension of DNA synthesis of overlapped oligo-nucleotides using PCR [30]. Emission spectra of the FP products were determined using a USB-4000 Spectrometer (Ocean Optics, Dunedin, FL, USA); the absorption spectra were measured using a Gene Spec V Spectrometer (Hitachi, Tokyo, Japan). Both fluorescence and absorption were measured three to six times for each protein except absorption of S2 (once). The absorption spectra were used as the approximate excitation spectra in this study.

## Results and Discussion

### Isolation of a novel *FP* gene

**Fig. 1:**
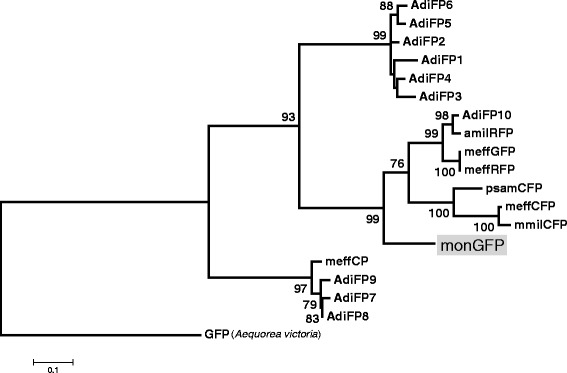
A phylogenetic tree based on the *FP* gene sequences. The tree was constructed using the maximum likelihood method. The bootstrap probability (>60) for each clade was obtained by 1,000 replicates and is shown next to each node. The evolutionary distances were computed using the p-distance method. All sites containing gaps and missing data were eliminated from the sequences used in the analysis. There were a total of 333 nucleotide sites in the final dataset. Evolutionary analysis was conducted in MEGA5. The *monGFP* is shown in gray. The scale bar represents 0.05 substitutions per site. *GFP* from *Aequorea victoria* was used as an outgroup. The accession numbers and species name for the *FP* sequences are as follows: *Acropora digitifera*, *AdiFP1* BR000962, *AdiFP2* BR000963, *AdiFP3* BR000964, *AdiFP4* BR000965, *AdiFP5* BR000966, *AdiFP6* BR000967, *AdiFP7* BR000968, *AdiFP8* AB698751, *AdiFP9* BR000969, *AdiFP10* BR000970; *Montipora millepora*, *meffCFP* DQ206381, *mmilCFP* DQ206392, *Montipora efflorescens*, *meffCP* DQ206377, *meffGFP* DQ206393, *meffRFP* DQ206379; *Psammocora* sp., *psamCFP* EU498721; and *Aequorea victoria GFP* M62653

**Fig. 2:**
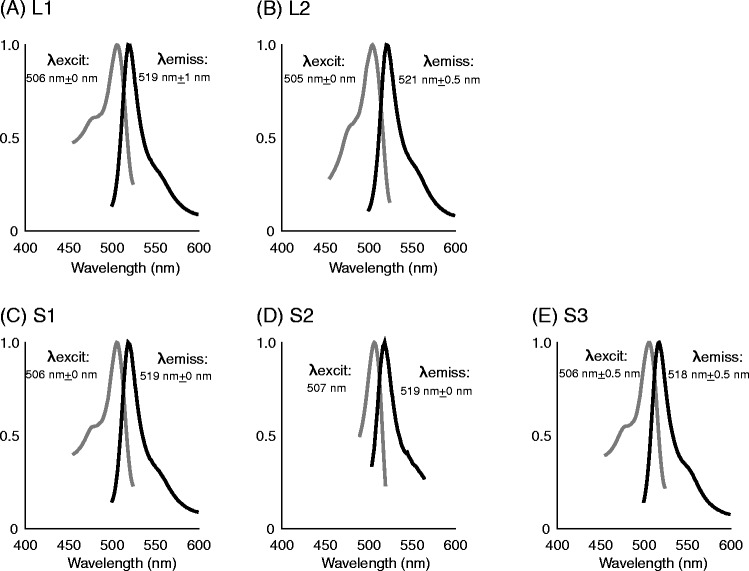
Excitation and emission spectra of monGFPs Excitation (gray line) and emission (solid line) spectra of the monGFPs from the variants L1 (**a**), L2 (**b**), S1(**c**), S2 (**d**), and S3 (**e**). Horizontal and vertical axes indicate wavelength in nanometers and normalized fluorescence amplitude, respectively

As a first step to isolate *FP* genes from *Montipora* sp., we designed a set of primers to amplify the *RFP* gene, as we expected that the *RFP* gene might be highly expressed in our reddish-colored specimen. We designed a set of degenerated primers (RFP_F2 and RFP_R) based on *amilRFP* (from *A. millepora*, accession number: AY646073) and *meffRFP* (from *M. efflorescens*, accession number: DQ206379) genes. We cloned and sequenced a PCR product amplified using these primers and cDNA of *Montipora* sp. as a template. We confirmed a single *FP*-like sequence showing high similarity (>80 %) with an *FP* nucleotide sequence (*meffGFP* from *M. efflorescens*, accession number: DQ206393) and protein sequences (Fig. S3a). This indicated that the newly isolated sequence was an *FP* family gene, which we tentatively named *monFP* (i.e., *FP* gene from *Montipora* sp. #5). To isolate the full-length coding region of *monFP*, we performed 5’ and 3’ RACE. Similar to known *FP* genes in other species, the coding region of *monFP* was 723 bp. We constructed a neighbor-joining phylogenetic tree using nucleotide sequences of the *monFP* together with other *FP* genes available from *Acropora digitifera (AdiFP1-AdiFP10*), *Acropora millepora* (*amilRFP), Montipora millepora,* (*mmilCFP*)*, Montipora effloresce* (*meffCP, meffCFP, meffGFP meffRFP*), and *Psammocora* sp. (*psamCFP*)*.* The *monFP* gene formed a monophyletic group with *AdiFP10, amilRFP, meffGFP, meffRFP, psamCFP, meffCFP, and mmilCFP* (Fig. 1). Within this group, *monFP* was distantly related to other *FP* genes, located in a basal position (Fig. 1). To elucidate the fluorescent class of the *monFP* gene, we cloned the *monFP* gene into an expression vector and measured the fluorescence of the protein product. As shown in Fig. 2e (the first measured sequence was named S3, see later section), the monFP protein emitted fluorescence with a maximum peak (λemiss) at 518 nm, indicating that monFP belongs to the GFP class [16, 17]. The wavelength distribution of fluorescence emitted from monFP also showed a shoulder at 550–570 nm (Fig. 2e); the fluorescence of monFP is thus perceived by eye as a yellowish color. From these results, we conclude that *monFP* is a novel fluorescent protein gene, which we have classified as a GFP [16, 17] and renamed *monGFP*. As shown in Fig. 1, although *monGFP* formed a monophyletic group with three *RFP* genes and one *GFP* gene (*meffGFP*), this group contained two different fluorescent color classes. One of the three amino acids (marked by asterisks in Fig. S3a) responsible for chromophore-formation of FPs was leucine in two GFPs, but aspartic acid in the other three RFPs. This leucine was unique to GFPs and the site may be responsible for the difference in the fluorescent color, representing the wavelength of emission light of monGFP.

### Alternative splicing and gene duplication of *monGFP*

**Fig. 3:**
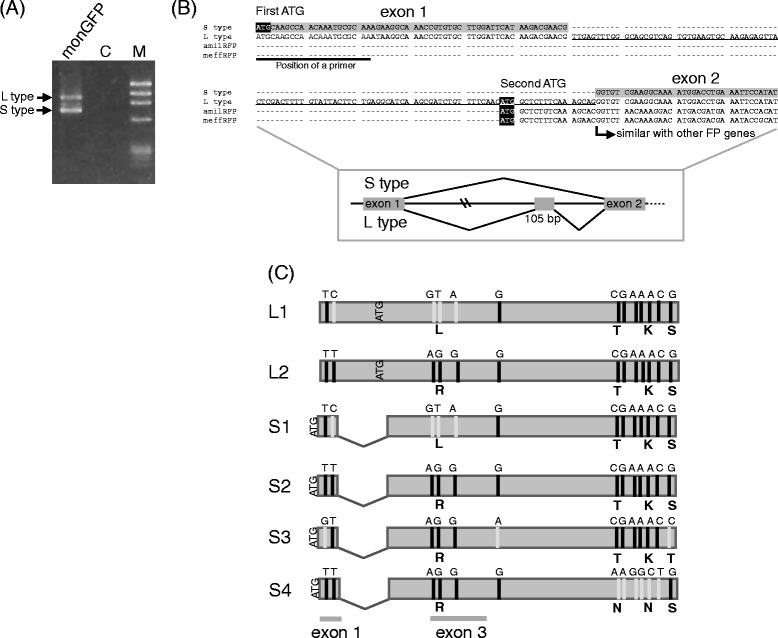
Splicing variants of *monGFP* (**a**) RT-PCR amplification of *monGFP* mRNAs from *Montipora* sp. M and C indicate molecular marker øX174/*H*aeIII and the negative control, respectively. The PCR products for each type of mRNA are marked by arrows. (**b**) A nucleotide alignment of the 5’ end of *monGFP* variants (M5_S type and M5_L type) with *RFP*s (*amilRFP* and *meffRFP*). The first and second initiation codons (ATG) are highlighted in black. The additional sequence in M5_L type cDNA is underlined. Exons 1 and 2 are shown in gray. The position of upstream primer (M5_F1) is shown by bold line. Schematic representation of the position of the additional sequence and splicing of S and L types is shown in lower panel. (**c**) Schematic representation of *monGFP* variants. Nucleotide differences among variants are shown by gray (minor alleles) and black (major alleles) bars. The alphabetical characters indicate nucleotides (above the box) and amino acids (below the box). The first and second initiation codons are shown as “ATG” in the box. The positions of exons 1 and 3 are shown at the bottom

To amplify the full-length *monGFP* from cDNA of *Montipora* sp. by PCR, we designed primers (M5_F1 and M5_R1) at both ends of the *monGFP* sequence. The PCR products showed a band with expected size (short type) and an unexpected one (long type) in the electrophoresis image shown in Fig. 3a. We cloned and sequenced both products and found that the long type contained an additional 105 bp (Fig. 3b). The reading frame of additional sequence is different from the original short type and it contains nonsense mutations if the translation starts from the same initiation codon as the short product. Rather, the long type uses the same initiation codon as *amilRFP* and *meffRFP* (Fig. 3b). The translation of the long type cDNA may start from the second initiation codon and yield a smaller protein than the short type cDNA yields. We named the product corresponding to the long cDNA type as L type (encoding small protein) and that to the short type as S type (encoding large protein). In total, we identified at least two long type (L1 and L2) and four short type (S1, S2, S3, and S4) cDNA sequences based on the nucleotide differences (Fig. 3c). The downstream region of the additional segment was identical between L1 and S1, and between L2 and S2 (Fig. 3c), indicating that the sequence pairs may be transcribed from the same gene and may have different 5’ end sequences attributed to alternative splicing.

**Fig. 4:**
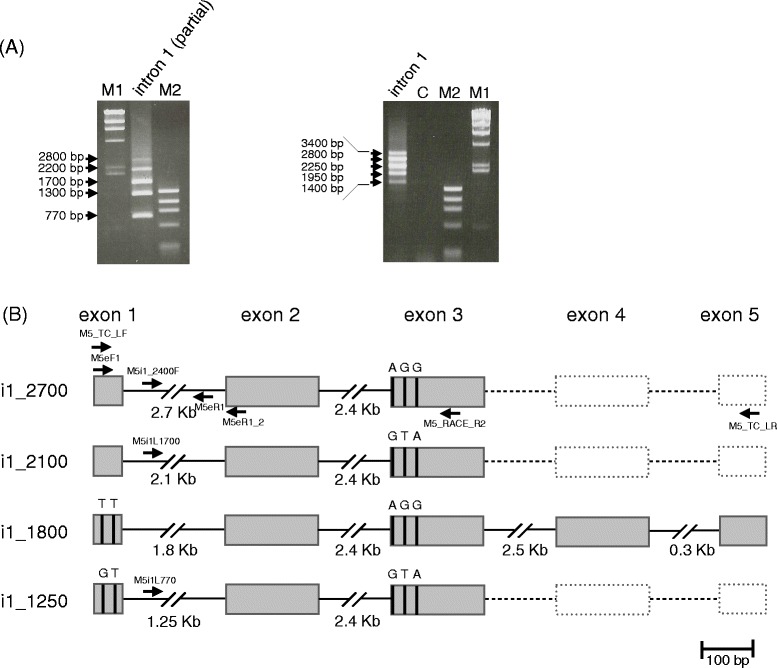
The structure of *monGFP* genes (**a**) PCR amplification of partial (left) and full-length (right) of *monGFP* intron 1 from the genomic DNA of *Montipora* sp.. M1, M2 and C indicate molecular markers λ/*Hin*dIII, øX174/*H*aeIII and negative control, respectively. The PCR products of each intron 1 sequence are marked by arrows. **b** Schematic representation of the structure of *monGFP* genes. Nucleotide differences among variants are shown by bars. The alphabetical characters indicate nucleotides (above the boxes). Dotted boxes indicate unidentified exons. The approximate lengths of introns are shown below the introns. Positions of primers used in PCR amplification to determine genomic structures are indicated by arrows

To confirm the alternative splicing, we determined exon-intron structure of this gene. We amplified a DNA fragment from exon 1 through to the additional 105 bp (Fig. 3b) of *monGFP* from genomic DNA using a primer set of M5eF1 and M5eR1. Surprisingly, the PCR products showed at least five bands with different lengths (Fig. 4a left panel: approximately 770, 1,300, 1,700, 2,200, and 2,800 bp). We cloned and sequenced four bands, but could not clone the longest band due to an insufficient quantity of amplified DNA. We designed primers specific to each DNA fragment and the region showing similarity with *amilRFP* and *meffRFP* (“similar with other FP genes” in Fig. 3b) to amplify DNA fragments, including additional 105 bp of *monGFP* from its genome, by using these specific primers and M5eR1_2. We determined the sequences of the PCR products and combined with the sequences of 770, 1,300, 1,700, and 2,200 bp DNA fragments. The combined sequences were 1,249, 1,818, 2,114, and 2,679 bp in length and the presence of the sequences in the genome was confirmed by PCR amplified from genomic DNA using a primer set of M5eF1 and M5eR1_2 (Fig. 4a right panel: approximately 1,400, 1,950, 2,250, 2,800, and 3,400 bp, including exon sequences at both ends). The 5’ and 3’ ends of the sequences of four products with different length were similar to each other and were connected with sequences in cDNA at the both ends. Thus, all four nucleotide sequences were intron 1 of *monGFP*. The nucleotide sequence 550 bp upstream from 5’ end of exon 2 (Fig. 3b lower panel) was identical to the additional sequence in L type cDNAs, suggesting that L type cDNAs were generated by alternative splicing. The length differences among intron 1 sequences were due to insertions or deletions in the upstream part from the additional 105 bp in the intron. The four intron 1 sequences of different sizes are likely to be derived from different genes. Based on the size of intron 1, products were named as the genes *i1_1250, i1_1800, i1_2100*, and *i1_2700* (Fig. 4b). To obtain the entire gene sequences, we designed a set of primers (M5_TC_LF and M5_TC_LR) at the 5’ and 3’ ends of the *monGFP* gene and amplified the full-length gene using the genomic DNA of *Montipora* sp. as a template. However, we obtained an only 7 kb PCR product and sequenced this product and identified the five exons and both ends of each of four introns. (Fig. 4b, *i1_1800*). The sequence of intron 1 of this PCR product was 1,950 bp, shown in Fig. 4a right panel, correspond to *i1_1800*. Next, we designed primers specific to the *i1_1250, i1_2100*, and *i1_2700* intron 1 sequences, and amplified the sequences from the genomic DNA between intron 1 and exon 3 using the primers specific to intron 1 and M5_RACE_R2. We obtained four products with nearly the same length (4 kb) and deduced the length of the sequences of intron 2 for each gene (Fig. 4b). We also tried to amplify the sequences between intron 1 and exon 5 using specific primers to the intron 1 of *i1_1250*, *i1_2100*, and *i1_2700* genes with combination of the exon 5 primer (M5_TC_LR), but could not obtain amplified products. The introns 3 and/or 4 of these genes might be too long to be amplified by using PCR. We then determined the sequences of exon 1 to exon 3 of each *monGFP* gene, except the sequences of exon 1 from *i1_2100* and *i1_2700* genes. We could not determine these exon 1 sequences by direct sequencing, probably due to variations of short indels in intron 1 of PCR products. In the sequences of exon 1 to exon 3, the variation observed in each cDNA sequence was confirmed. Based on the exons 1 and 3 sequence types, only one pair, S2 and L2 variants with exon1 (TT) exon3 (AGG) matched with *i1_1800* gene with exon1 (TT) exon3 (AGG) (Fig. 3c and 4b). *i1_1250* gene with exon1 (GT) exon3 (GTA) did not match with other variants (Fig. 3c and 4b), possibly due to low or no transcription from this gene in an adult *Moptipora* sp.. Based on the exon 3 sequence types, S1 and L1 variants with exon3 (GTA) matched with i1_2100 gene with exon3 (GTA) (Fig. 3c and 4b). S3 and S4 variants with exon3 (AGG) matched with only one gene, i1_2700 with exon3 (AGG) (Fig. 3c and 4b). It is possible that there is another *monGFP* gene with intron 1 sequence 3,400 bp in length (shown in Fig. 4a right panel), and that one of the S3 and S4 variants may be transcribed from this gene. From these results, we conclude that *monGFP* genes have undergone recent gene duplications that generated at least four genes, and six different transcripts of these are transcribed from *monGFP* genes.

To examine the functions of different genes and splice variants, we generated monGFP proteins from variants L1, L2, S1, and S2 to measure fluorescence. As shown in Fig. 4, the maximum excitation spectra (λexcit) and the maximum emission spectra (λemiss) of all monGFPs ranged from 505 nm to 507 nm, and from 518 nm to 521 nm, respectively. These results suggest that monGFPs translated from duplicated genes and alternatively spliced mRNAs exhibit nearly identical functions. The difference in λemiss between L1 and L2 monGFPs was 2 nm (Fig. 4a and b) and was attributed to a single amino acid replacement in exon 3 (Fig. 3c). The amino acid sequence from exon 2 to 5 of each variant pair of L1 and S1, or L2 and S2, was identical (Fig. 3c), but those of N terminal regions of S and L types were completely different (Fig. S3b). The fluorescence emitted from monGFPs of L1 and S1 was equivalent (Fig. 4a and c), and that of L2 and S2 was different by 2 nm (Fig. 4b and d). Although the length and amino acid sequences of the N terminal were different between S and L types, the differences in proteins attributed to alternative splicing do not cause a large difference in the wavelength of emitted fluorescence. FPs translated from different gene copies have been strongly conserved in the function of fluorescence. The analysis of the ratio of substitution rates at non-synonymous and synonymous sites (dN/dS) showed negative selection acting on this gene family (0.18 ± 0.01). These results suggest that *monGFP* genes have evolved under purifying selection.

The question of the biological role of gene duplication and alternative splicing of *monGFP* genes thus arises. One possible role is in increasing the expression of proteins with the same function. In *A. millepora*, the copy number variation of *amilFP597* with a particular promoter type is correlated with red pigment concentration. The higher expression of *amilFP597* has a photoprotective role to reduce photodamage to zooxanthellae under acute light [4]. The other possibility is differential expression of splicing variants in different tissues. Although the fluorescent spectra were nearly identical, the increased gene copy number could increase the diversity in gene expression in different tissues [31] if each gene in the family is regulated independently. Since the quantity of the PCR products were nearly the same in the electrophoresis gel (Fig. 3a, S and L types), the expression levels of the two types of variants were roughly comparable, suggesting that both types may be functional in live corals. The two types of variants might have tissue-specific functions, similar to the muscle-specific roles of the splicing variants of the *Mef2D* gene [32].

### Gene duplication of the *AdiFP10* paralog

**Fig. 5:**
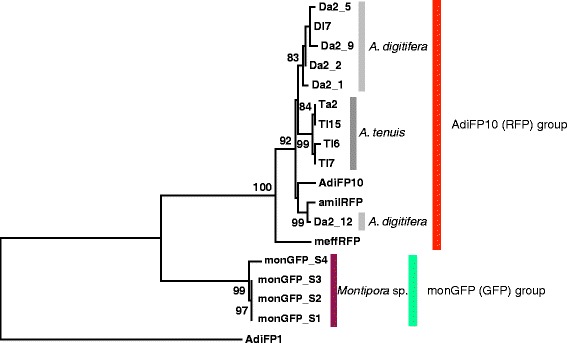
Phylogenetic relationships among paralogs of *AdiFP10* A phylogenetic tree was constructed by the maximum likelihood method using *FP* gene nucleotide sequences. The bootstrap probability for each clade was obtained by 1,000 replicates and is shown next to each node. The evolutionary distances were computed using the p-distance method. All sites containing gaps and missing data were eliminated from the sequences in the analysis. There were a total of 351 nucleotide sites in the final dataset. Evolutionary analysis was conducted in MEGA5. The scale bar represents 0.05 substitutions per site. *AdiFP1* was used as an outgroup. *A. digitifera* adult: Da, larvae: Dl, *A. tenuis* adult: Ta, and larvae: Tl

As shown in Fig. 1, *monGFP* forms a monophyletic clade with the other *RFP* and *GFP* genes. To examine whether genes in other species in this clade have undergone gene duplication, we designed a set of primers (AdiFP10_F2 and AdiFP10_R1) based on the *AdiFP10* sequence to identify other *FP* genes expressed in *A. digitifera* and *A. tenuis*. We extracted RNA and synthesized cDNA separately from a single adult and large samples (over 100) of larvae from each species. We determined 97 sequences in total from the PCR products and isolated six and four distinct sequences from *A. digitifera* and *A. tenuis*, respectively. We constructed a phylogenetic tree using the isolated sequences, together with *monGFP* genes, *AdiFP10*, and two *RFP* genes, using *AdiFP1* as an outgroup (Fig. 5). In this tree, the *A. tenuis* sequences formed a monophyletic group, whereas *A. digitifera* sequences were located in two different clades and the clades are different from a *monGFP* clade (Fig. 5). Moreover, five different sequences (*Da2_1*, *Da2_2*, *Da2_5*, *Da2_9*, and *Da2_12*) were isolated from a single adult individual of *A. digitifera*. Since *A. digitifera* is diploid [24], we estimated that at least three genes present in the genome, even if all five sequences are allelic variation. One of the five sequences, *Da2_12*, clustered with the *amilRFP* gene, and the others formed a monophyletic group (Fig. 5). This result suggests that a gene duplication event occurred before the divergence of *Acropora* species, and additional duplication occurred in the *A. digitifera* lineage, if no gene conversion is assumed in this species. In *A. tenuis*, three sequences were obtained from larvae cDNA, and we are not sure that whether the sequence variation was due to gene duplication events or allelic variation. These results suggest that there are at least three paralogs of *AdiFP10* in *Acropora* species.

In this study, we showed that diversification of *FP* genes in Scleractinia occurred by gene duplication events and alternative splicing. We found at least four *monGFP* genes in the genome of *Montipora* sp., each with a similar function in terms of emitted fluorescence. Regarding the wavelength of fluorescence, *monGFP* genes have evolved without change in function even though the amino acid sequences of the first exon in different splicing variants are completely different. The similar function of duplicated *monGFP* may be attained through both purifying selection and homogenization by gene conversion, although the evidence supporting gene conversion in *monGFP* remains unclear. One of possible biological causes for the homogenization might be a requirement of increasing amount of the protein. Although the conserved evolution of the function of FPs is not well studied, this mode of evolution may be an important aspect with regard to FPs. When corals regulate the expression level of FPs to adapt or acclimatize to different environments, such as various light stresses [4], the functionally conserved multi-copy *FP* genes may be advantageous due to their ability to greatly change FP expression levels. On the other hand, gene duplication events and the acquisition of new functions has also been suggested in the FP family [2]. *AdiFP10* paralogs have diversified in *Acropora* species (Fig. 5). Although we did not measure the emission spectra of the newly identified paralogs, *amilRFP* and *meffRFP* possess distinct fluorescent spectra, with emission peaks at 593 nm and 576 nm, respectively [17], suggesting that *AdiFP10* paralogs and other *RFP* genes in *Acropora* species may have diversified with respect to their functions, *i.e.*, fluorescence. Thus, these *FP* genes are consistent with divergent evolution, as suggested previously [2]. Our results regarding evolution of *FP* genes are consistent with various modes of evolution. These differences might reflect variation in the biological roles of different FPs. Further detailed evolutionary analysis of *FP* genes may reveal the roles of FPs in live corals.

## Additional file

Additional file 1: Figure S1. Phylogenetic relationships of *Montipora* sp. #5 A phylogenetic tree was constructed by the neighbor-joining method using *COI* sequences. The bootstrap probability for each clade was obtained by 1,000 replicates and is shown next to each node. The evolutionary distances were computed using the p-distance method. All sites containing gaps and missing data were eliminated from the sequences in the analysis. There were a total of 610 nucleotide sites in the final dataset. Evolutionary analysis was conducted in MEGA5. The scale bar represents 0.05 substitutions per site. Accession numbers of *COI* sequences from *Montipora* species are listed after species name. **Figure S2.** Positions of primers used in this study. Positions of primers are indicated by arrows above or under the schematic representation of the structure of *monGFP* genes. The positions of primers on intron 1 are indicated in the box. **Figure S3.** Amino acid alignments of *FP* genes. (A) An amino acid alignment of *FP* genes. Identical amino acids among the three sequences are shown in gray. Chromophore-forming tri-peptides are marked by asterisks. The accession numbers and species name for the *FP* sequences are as follows: *Acropora digitifera*, *AdiFP1* BR000962, *AdiFP2* BR000963, *AdiFP3* BR000964, *AdiFP4* BR000965, *AdiFP5* BR000966, *AdiFP6* BR000967, *AdiFP7* BR000968, *AdiFP8* AB698751, *AdiFP9* BR000969, *AdiFP10* BR000970; *Montipora millepora*, *mmilCFP* DQ206392, *Montipora efflorescens*, *meffCFP* DQ206381, *meffCP* DQ206377, *meffGFP* DQ206393, *meffRFP* DQ206379; and *Psammocora* sp., *psamCFP* EU498721. (B) An amino acid alignment of N terminal of S type and L type. Identical amino acids among the three sequences are shown in gray. An identical region is shown by dotted lines.
