# Supplementary material for: Genetic diversity of fluorescent protein genes generated by gene duplication and alternative splicing in reef-building corals
Source: Zoological Lett. 2015 Jul 21;1:23. doi: 10.1186/s40851-015-0020-5 (PMC4657232; doi:10.1186/s40851-015-0020-5)
Supplement: Additional file 1: Figure S1. — Phylogenetic relationships of Montipora sp. #5 A phylogenetic tree was constructed by the neighbor-joining method using COI sequences. The bootstrap probability for each clade was obtained by 1,000 replicates and is shown next to each node. The evolutionary distances were computed using the p-distance method. All sites containing gaps and missing data were eliminated from the sequences in the analysis. There were a total of 610 nucleotide sites in the final dataset. Evolutionary analysis was conducted in MEGA5. The scale bar represents 0.05 substitutions per site. Accession numbers of COI sequences from Montipora species are listed after species name. Figure S2. Positions of primers used in this study. Positions of primers are indicated by arrows above or under the schematic representation of the structure of monGFP genes. The positions of primers on intron 1 are indicated in the box. Figure S3. Amino acid alignments of FP genes. (A) An amino acid alignment of FP genes. Identical amino acids among the three sequences are shown in gray. Chromophore-forming tri-peptides are marked by asterisks. The accession numbers and species name for the FP sequences are as follows: Acropora digitifera, AdiFP1 BR000962, AdiFP2 BR000963, AdiFP3 BR000964, AdiFP4 BR000965, AdiFP5 BR000966, AdiFP6 BR000967, AdiFP7 BR000968, AdiFP8 AB698751, AdiFP9 BR000969, AdiFP10 BR000970; Montipora millepora, mmilCFP DQ206392, Montipora efflorescens, meffCFP DQ206381, meffCP DQ206377, meffGFP DQ206393, meffRFP DQ206379; and Psammocora sp., psamCFP EU498721. (B) An amino acid alignment of N terminal of S type and L type. Identical amino acids among the three sequences are shown in gray. An identical region is shown by dotted lines. [file 40851_2015_20_MOESM1_ESM.pdf]

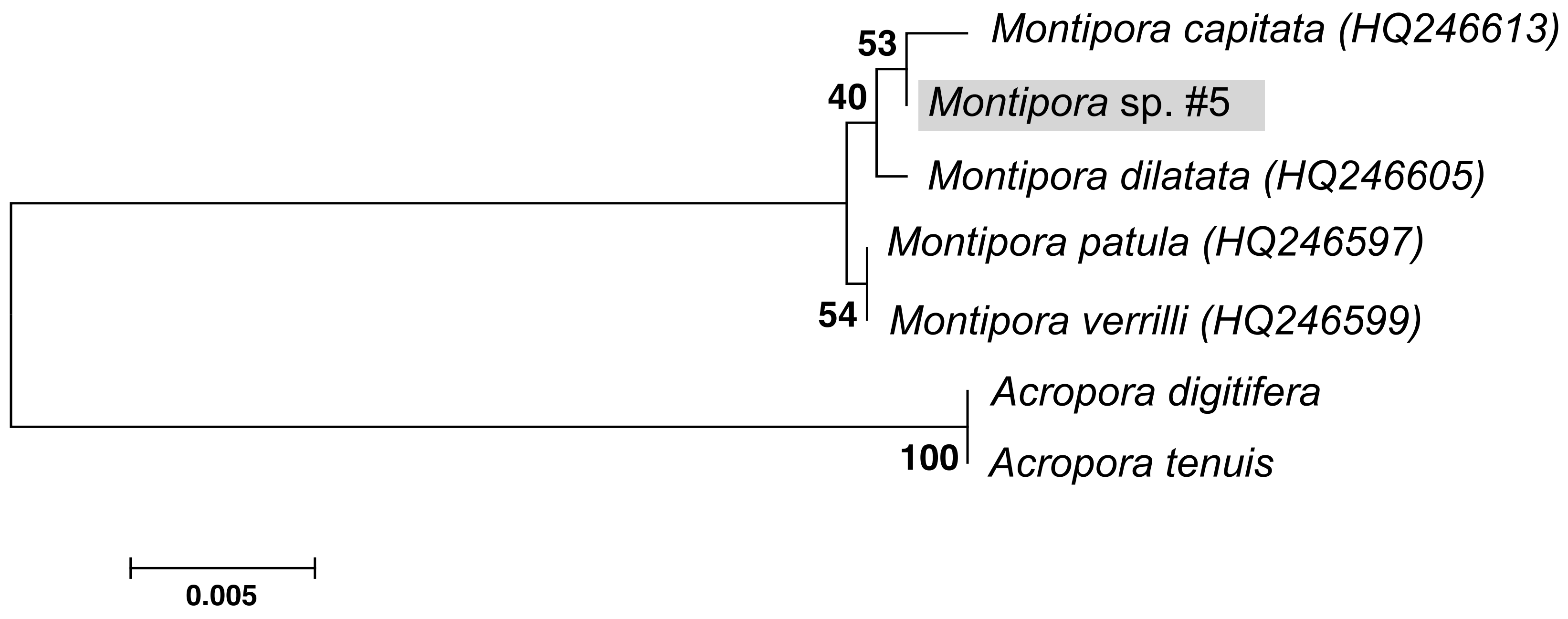

Montipora capitata (HQ246613)
53
Montipora sp. #5
40
Montipora dilatata (HQ246605)
Montipora patula (HQ246597)
54
Montipora verrilli (HQ246599)
Acropora digitifera
100
Acropora tenuis
0.005

Figure S1

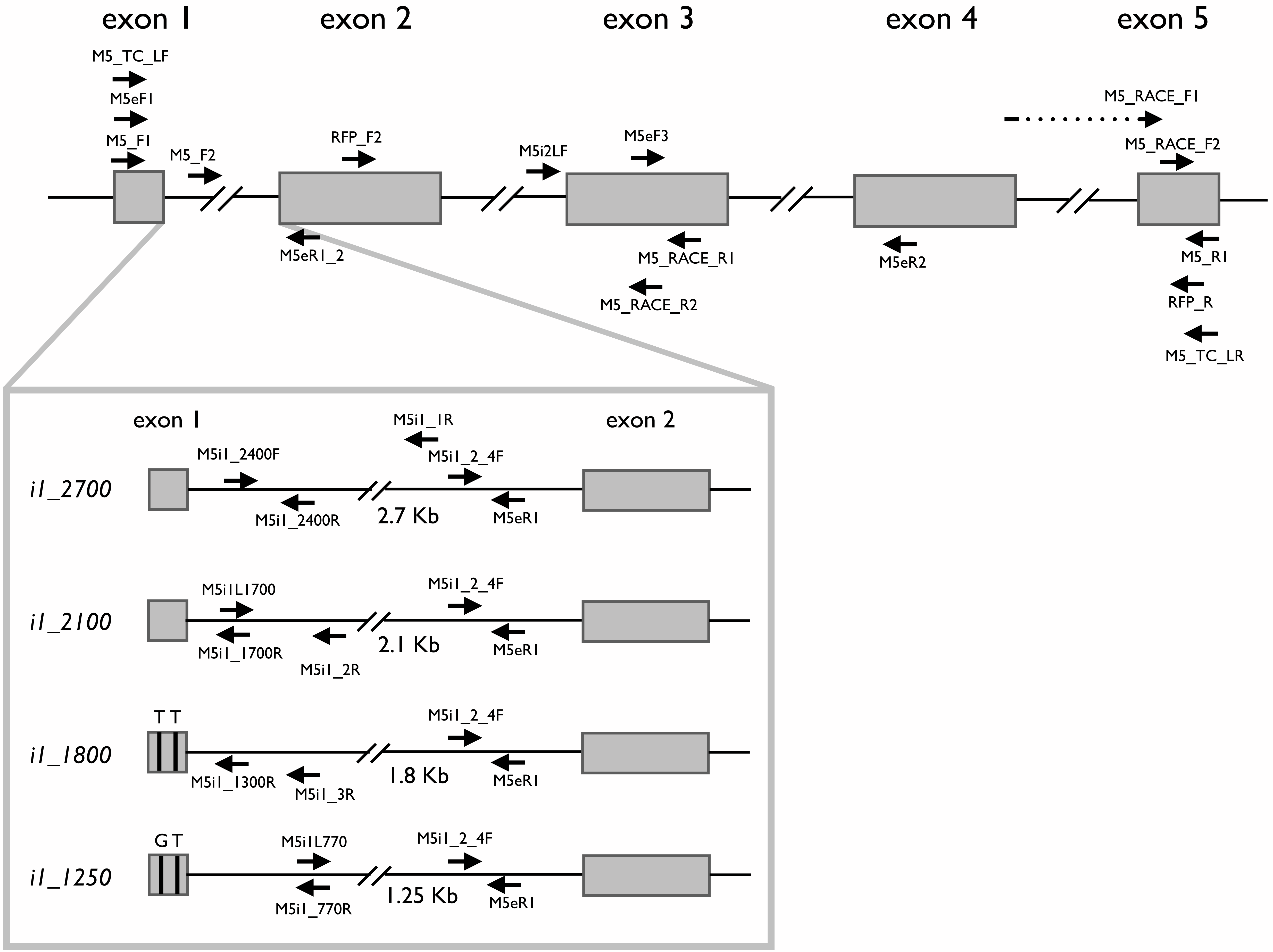
exon 1
exon 2
exon 3
exon 4
exon 5
M5_TC_LF
M5eF1
M5_F1
M5_F2
RFP_F2
M5i2LF
M5eF3
M5_RACE_F1
M5_RACE_F2
M5eR1_2
M5_RACE_R1
M5_RACE_R2
M5eR2
M5_R1
RFP_R
M5_TC_LR
exon 1
exon 2
M5i1_1R
M5i1_2400F
M5i1_2_4F
il_2700
M5i1_2400R
2.7 Kb
M5eR1
M5i1L1700
M5i1_2_4F
il_2100
M5i1_1700R
M5i1_2R
2.1 Kb
M5eR1
T T
M5i1_2_4F
il_1800
M5i1_1300R
M5i1_3R
1.8 Kb
M5eR1
G T
M5i1L770
M5i1_2_4F
il_1250
M5i1_770R
1.25 Kb
M5eR1

Figure S2

(A)

```
N-terminal
meffGFP ---------- ----MALSKN GVKDRMKLKF HMEGSVNGHE FTIKGEGTGQ PYEGTQS-IQ LRVEKGGPLP FSVDILSAVF LYGNRVFTKY PQDLVDYFKN SCP-AGYTWQ RSFLFEDGAV CTASADITVS
meffRFP ---------- ----MALSKN GLTKNMTTKY RMEGCVDGHK FVITGDGIGD PFEGKQTSID LCVVEGGPLP FSEDILSAVF DYGNRVFTKY PQDLVDYFKN SCP-AGYTWQ RSFLFEDGAV CTASADITVS
AdiFP10 ---------- ---------- ---------- -MEGSVDGHK FVITGHGNGN PFEGKQT-MN LCVVEGGPLP FSEDILSAVF DYGNRVFTEY PQGMVDFFKN SCP-AGYTWQ RSLLFEDGAV CTASADITVS
amilRFP ---------- ----MALSKH GLTKDMTMKY HMEGSVDGHK FVITGHGNGN PFEGKQT-MN LCVVEGGPLP FSEDILSAAF DYGNRVFTEY PQGMVDFFKN SCP-AGYTWH RSLLFEDGAV CTTSADITVS
monGFP  MQANKCAKKA NRVLGFIRRT GVEGKMDLKF HMEGSVNGHE FTIKGEGTGQ PYEGTQC-IQ LRVEKGGPLP FSVDILSAVF LYGNRCITKY PRGIVDYFKN SCP-DGYKWE RSFLFEDGAV CTACADIRVS
psamCFP ---------- ----MASTKN VLPNMMTLTY HMEGSVNGHN FEIIGEGTGN PKEGKHT-IT LQVVKGGPLP FSVDILSTVF QYGNRCFTKY PPNTVDYFKN SCP-PGYTFE RSFLYEDGAV CTASGDITLS
mmilCFP ---------- ---------M ALPKQMKLTY HMEGTVNGHF FIIKGEGGGE PYEGTHT-IK LQVVEGSPLP FSPDILSTVF QYGNRCFTKY PPNIVDYFKN SCSGGGYTFG RSFLYEDGAV CTASGDITLS
meffCFP ---------- ----MALSKQ SLPSDMKLIY HMDGNVNGHS FVIKGEGEGK PYEGTHT-IK LQVVEGSPLP FSADILSTVF QYGNRCFTKY PPNIVDYFKN SCSGGGYKFG RSFLYEDGAV CTASGDITLS
                                                                                           ***

meffGFP VEENCFYHES KFHGVNFPAD GPVMKKMTTN WEPSCEKITP IPNEGILKGD VTMFLLLKDG GRYRCQFDTV YKAKSDPKTI MMPDWHFIQH KLNREDRSDA KHQKWRLVEN AIAYRSTLS
meffRFP VEENCFYHES KFHGVNFPAD GPVMKKMTTN WEPSCEKITP IPNEGILKGD VTMFLLLKDG GRYRCQFDTV YKAKSDPKTI MMPDWHFIQH KLNREDRSDA KHQKWRLVEN AIAYRSTLS
AdiFP10 VEENCFYHES QFHGVNFPAD GPVMKKITTN WEPSCEKIIP VPRQGILKGD IAMYLLLKDG GRYRCQFDTV YKANSDPKK- -MPGWHFIQH KLIREDRGDA KNQKWQLVEH AVASRSALPG
amilRFP VEENCFYHNS KFHGVNFPAD GPVMKKMTTN WEPSCEKIIP VPRQGILKGD IAMYLLLKDG GRYRCQFDTI YKAKSDPKE- -MPEWHFIQH KLTREDRSDA KNQKWQLVEH AVASRSALPG
monGFP  VEDNCFYHES KFCGVNFPAD GPVMTKVTTG WEPSSEKMVP C--VGILNGD VTMFLLLKDG TRYRCQFHST YKAKTGPKE- -MPDFHFVEH KIVRKDLG-G RDQKWQLVGS SSASASSL
psamCFP DDKASFHHKS KFFGVNFPDD GPVMKKKTTD WEPSCEKMTP S--GKTLKGD VIEFLLLEGG GRYKCQFHTV YRAKTEPKR- -MPEFHFVQH KLTRTDVSDP LKQQWQLTED AAACESCFHK
mmilCFP SDKSSFEHKS KFLGVNFPAD GPVMKKETTN WEPSCEKMTP N--GMTLIGD VTEFLLKKDG KRYKCQFHTF HDAKEKSRNM PMPDFHFVQH EIERKDLP-G PMQTWQLTEH AAACKNCFTE
meffCFP ADKKSFEHKS KFLGVNFPAD GPVMKKETTN WEPSCEKMTP N--GMTLIGD VTGFLLKEDG KRYKCQFHTF HDAKDKSKKM PMPDFHFVQH KIERKDLP-G SMQTWRLTEH AAACKTCFTE
                                                                                                                     C-terminal
```

(B)

```
           N-terminal                                      Identical region
S type   MQANKCAKKANRVLGFIRRTGVEGKMDLKFH..............................................
L type   M--------------ALSKQGVEGKMDLKFH..............................................
                                                                              C-terminal
```

Figure S3
